# Refining Glioblastoma Surgery through the Use of Intra-Operative Fluorescence Imaging Agents

**DOI:** 10.3390/ph15050550

**Published:** 2022-04-29

**Authors:** Oluwakanyinsolami Netufo, Kate Connor, Liam P. Shiels, Kieron J. Sweeney, Dan Wu, Donal F. O’Shea, Annette T. Byrne, Ian S. Miller

**Affiliations:** 1 Precision Cancer Medicine Group, Department of Physiology and Medical Physics, Royal College of Surgeons in Ireland, 2, D02 YN77 Dublin, Ireland; kanyisolanetufo@rcsi.ie (O.N.); kateconnor@rcsi.com (K.C.); liamshiels@rcsi.com (L.P.S.); kieronsweeney@beaumont.ie (K.J.S.); annettebyrne@rcsi.com (A.T.B.); 2 National Centre for Neurosurgery, Beaumont Hospital, 9, D09 V2N0 Dublin, Ireland; 3 Department of Chemistry, Royal College of Surgeons in Ireland (RCSI), 2, D02 YN77 Dublin, Ireland; danwu@rcsi.com (D.W.); donalfoshea@rcsi.com (D.F.O.); 4 National Pre-Clinical Imaging Centre (NPIC), 2, D02 YN77 Dublin, Ireland

**Keywords:** glioblastoma, fluorescence guided surgery, 5-ALA, fluorescein, NIR-AZA

## Abstract

Glioblastoma (GBM) is the most aggressive adult brain tumour with a dismal 2-year survival rate of 26–33%. Maximal safe resection plays a crucial role in improving patient progression-free survival (PFS). Neurosurgeons have the significant challenge of delineating normal tissue from brain tumour to achieve the optimal extent of resection (EOR), with 5-Aminolevulinic Acid (5-ALA) the only clinically approved intra-operative fluorophore for GBM. This review aims to highlight the requirement for improved intra-operative imaging techniques, focusing on fluorescence-guided imaging (FGS) and the use of novel dyes with the potential to overcome the limitations of current FGS. The review was performed based on articles found in PubMed an.d Google Scholar, as well as articles identified in searched bibliographies between 2001 and 2022. Key words for searches included ‘Glioblastoma’ + ‘Fluorophore’+ ‘Novel’ + ‘Fluorescence Guided Surgery’. Current literature has favoured the approach of using targeted fluorophores to achieve specific accumulation in the tumour microenvironment, with biological conjugates leading the way. These conjugates target specific parts overexpressed in the tumour. The positive results in breast, ovarian and colorectal tissue are promising and may, therefore, be applied to intracranial neoplasms. Therefore, this design has the potential to produce favourable results in GBM by reducing the residual tumour, which translates to decreased tumour recurrence, morbidity and ultimately, mortality in GBM patients. Several preclinical studies have shown positive results with targeted dyes in distinguishing GBM cells from normal brain parenchyma, and targeted dyes in the Near-Infrared (NIR) emission range offer promising results, which may be valuable future alternatives.

## 1. Introduction

Glioblastoma (GBM) is the most common primary malignant brain tumour in adults [1,2]. The current standard of care (SOC) comprises maximum surgical resection followed by radiotherapy, with concomitant adjuvant Temozolomide (TMZ) chemotherapy [3]. Despite multimodal SOC, diagnosed patients have a dismal 2-year survival rate of just 26–33% [2]. Tumour resection serves a vital role to improve patient outcome via tumour debulking, cytoreduction and reduction of mass effect, and has been proven to significantly increase PFS [4,5]. The extent of resection (EOR) is the major determinant of surgical success [2], with complete resection of the detectable tumour (CRDT) the primary goal [4,5]. Complete resection (CR) of the contrast-enhancing tumour is associated with significantly improved overall survival (OS) among GBM patients (4.1 months OS vs. 1.8 months OS with partial resection) [3,6]. However, CR is virtually impossible. Thus, maximal safe surgical resection is a complex goal [4].

Several studies have assessed the impact of gross total resection (GTR) of the contrast-enhancing tumour on OS of GBM patients, while considering other predictive variables such as age, Karnofsky Performance Scale (KPS) score and absence of necrosis [4,7,8,9]. Additionally, factors such as the Ki-67 proliferation index (>20%) and high EGFR expression have been shown to be associated with poor overall survival [10,11,12]. An early study of *N* = 416 GBM patients sought to determine an association between EOR and survival time. Here, Lacroix et al. demonstrated that resection of 89% of enhancing tumours identified on T1 weighted MRI significantly improved OS (median OS 10.9 months) [8]. Indeed, the greatest improvement was observed in cases where >98% of contrast enhancing tumour was removed, resulting in a median OS of 13 months [8]. More recently, two additional studies [7,9] have demonstrated a significant survival benefit associated with extensive EOR. Sanai et al. showed that in *N* = 500 GBM patients, the subtotal EOR of 78% results in a significantly improved OS in newly diagnosed GBM, with aggressive EOR of >96% resulting in a further improved median OS of 12.2 months [7]. Finally, in a study of *N* = 1229 GBM patients, Li et al. demonstrated a 5.4 month increase in median OS, coupled with a 6% decrease in postoperative complications in patients who underwent CR of the T1 contrast-enhancing tumour [9]. Recently, several studies have shown that metabolic positron emission tomography (PET) may also support pre-operative planning, and aid in maximising EOR [13,14,15]. For example, a recent trial (NCT00006353) has implemented ^11^C-methionine (MET) to aid in the definition of tumour volume and support improved RT planning [13]. Here, ^11^C-MET identified tumour regions that were likely to recur, moreover the ^11^C-MET enhanced regions indicated where greater margins of resection would be beneficial. A further study, which compared MRI contrast enhancement with ^18^F-FDG and ^11^C-MET as applied to surgical planning, found that the use of pre-operative PET was associated with an increased survival in GBM patients compared with tumour resection based on MRI alone [14].

Nevertheless, the surgical resection of GBM is not curative [7,16,17]. Indeed, it remains an ongoing clinical challenge to intra-operatively delineate gliomas from normal brain tissue [16], where in some cases, resection of GBM tumours is regrettably associated with significant neurological deficit [6,16,18]. The use of stereotactic pre-operative and intraoperative imaging assists in the delineation of the brain tumour interface. Currently, neuro-navigation, a computerised technique used in the localising of tumour material in the brain, is vital in pre-operative planning for surgical resection. However, these images become invalidated as brain tissue shifts during resection and debulking [17]. Neuro-navigation systems may, however, be improved via implementation of intra-operative MRI (iMRI), intra-operative Ultrasound (iOUS), and Contrast Enhanced Ultrasound (CE-US) [4,5,19]. Indeed, these technologies remain essential in intra-operative guidance today notwithstanding limitations in their capacity to accurately identify all residual and invasive tumour material. In this context, tumour recurrence is usually inevitable.

Notwithstanding, there exists a significant need for improved intra-operative guidance to maximise the extent of resection, and ultimately improving patient prognosis [16]. To address this, fluorescence probes may be introduced to illuminate tumour margins where traditional white light imaging fails to delineate tumour-normal brain margins [4,6,18]. Indeed, fluorescence-guided surgery (FGS) aims to improve visualisation of tumour cells within the surgical field, particularly in the neuro-oncology context where diffuse and invasive tumour margins persist [18]. Overall, FGS provides the opportunity to improve EOR and realise the benefits of resection beyond the limitations of equivocal margins, either radiographically or under white light [4,16].

To date, several fluorophores have reached clinical trial (summarised in [Table 1]). Many of these fluorophores may also be used in combination, with improvements in GTR shown when using multiple fluorescence imaging agents in unison [4]. Additional benefits of FGS include affordable price, wider accessibility, and an absence of ionising radiation [19]. The majority of fluorescence imaging agents emit light in the visible spectrum (400–700 nm) [19]. However, the more favourable wavelength for in vivo use is the near infrared (NIR) range (700–900 nm) [19], enabling deeper imaging and photo penetration of up to 8 mm through tissue [20,21]. Indeed, despite positive outcomes afforded by these agents, many agents afford only a 2-dimensional image, and the signal can frequently be obscured by overhanging tissue, blood, and haemostatic agents. Fluorophores may also be photobleached or destroyed by coagulation [22]. Nevertheless, utilisation of fluorophores may significantly improve EOR and ultimately patient outcome [12].

**Figure 1:**
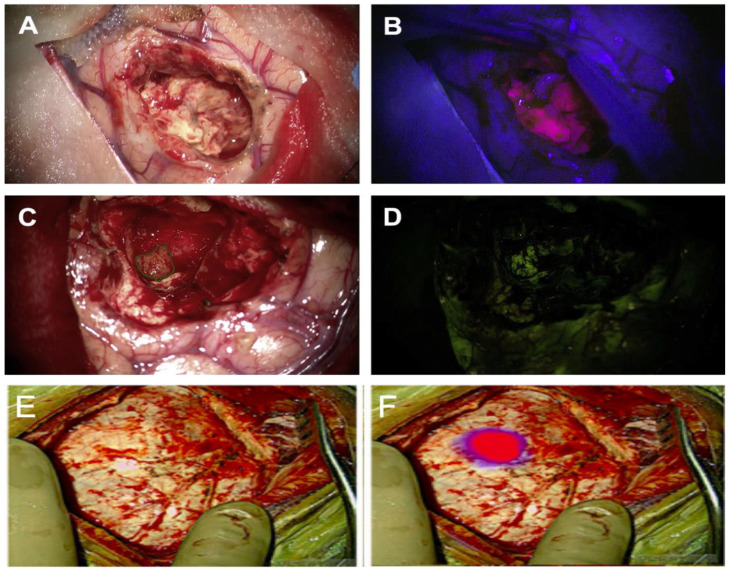
Examples of current fluorophores used in clinical practice. Representative images of fluorescence-guided resection of glioblastoma using (**A**,**B**) 5-ALA, (**C**,**D**) Fluorescein and (**E**,**F**) SWIG using white light (**A**,**C**,**E**), fluorescing light (**B**,**D**) or a white-light + NIR overlay (**F**). Images (**A**–**D**) reproduced with permission from Stummer et al. 2017. Fluorescence Imaging/Agents in tumour resection. *Neurosurg Clin. N. Am*. **2017**, *28*, 569–583. Images (**E** + **F**) reproduced with permission from Teng et al. 2021. *Neurosurg Focus.* **2021**, *50*, E4.

In the current review, we discuss the mechanisms and historical use of established fluorescent agents including Fluorescein Sodium, 5-Aminolevulinic Acid (5-ALA), Indocyanine Green, IRDye 800 CW, and Alkylphosphocholine analogues (APCs) (Figure 1). We further explore the evidence underpinning the development of novel fluorophores, which harbour potential to overcome limitations of current FGS. We describe the properties of these probes and reference pre-clinical trials that have yielded positive results. Building on previous reviews by Craig et al. (2016), Belykh et al. (2016) and Sun (2021), we expand on extensively researched probes such as Folate-targeted FGS, hypericin, and RGD Conjugated agents. We also highlight a novel class of switching fluorophores, NIR-AZA, and discuss their potential to overcome the shortcomings associated with currently established fluorophores. We postulate that the adaptation of these probes for use in GBM surgery is a promising area of translational research.

## 2. Established Fluorescent Agent Utilised in Surgery

### 2.1. Fluorescein Sodium

Fluorophores were first used in surgery in 1948, with Fluorescein sodium (FS), the sodium salt of the fluorescent organic dye Fluorescein [19], the first agent introduced to improve the identification of intra-operative brain tumours [4,22]. Accumulation of FS depends on a leaky blood-brain-barrier (BBB), and the regions in the brain of fluorescein accumulation correspond to those established by MRI contrast-enhancement [4,5,18]. During tumour resection, the BBB is disrupted, allowing extravasation of fluorescein [29] and results in fluorescence in non-tumour areas, for instance, following surgical manipulation, in surrounding oedematous but fundamentally healthy tissue. As a result, non-specific signals are frequently observed [18,30,31]. This non-tumour fluorescence results in fluorescein’s inability to serve as a tumour-specific marker and prevents clear-cut tumour resection [4,22]. Though an inexpensive method for intra-operative imaging, some studies have not shown an improvement in resection outcomes or improved survival of GBM patients using this agent [5,22]. In a study of *N* = 12 patients with high-grade glioma (HGG) who underwent FS-guided surgery, the fluorescein margins corresponded with that of gadolinium enhancement on MRI. Biopsy samples were taken and FS showed a sensitivity of 82.2% and specificity of 90% in distinguishing tumour cells from normal cortical tissue. The GTR of the enhancing tumour, as assessed by postoperative MRI, was achieved in every case. Infiltrating edges accumulate the least FS due to minimal BBB disruption [30], limiting its usefulness in enhancing tumour in resection surgeries. Moreover, as accumulation occurs in the extracellular space, the fluorescence emitted in dense tumours is restricted [29,30].

Nevertheless, in contrast to the above studies, the efficacy of FS for use in GBM resection has shown to be improved with the use of a surgical filter and this has yielded favourable results as seen in a phase II trial of 12 patients [31]. Of N = 20 biopsies performed at the resection margin (*N* = 5 patients), a sensitivity and specificity of 91% and 100% respectively of FS identifying tumour tissue was reported [31]. Indeed, in a recent study of 106 patients with GBM [32], GTR was seen in 84% of patients, thus displaying a great improvement compared to non-fluorescent guided surgery. Currently use of this probe for fluorescence-guided resection of glioma is still under consideration by the FDA [33]. 

### 2.2. 5-Aminolevulinic Acid (5-ALA)

The orally administered prodrug 5-ALA fluoresces slightly below the NIR spectrum and is currently the most widely used fluorophore in the clinic with peak fluorescence reached 6 h post administration [19]. The prodrug 5-ALA endogenously occurs in the heme synthesis pathway, whereby it is metabolised to Protoporphyrin IX (PpIX) in the mitochondria. Due to the reduced ferrochelatase activity of malignant cells, PpIX uptake is significantly increased in malignant tissue compared to normal tissue [4,5,18,19,34]. The 5-ALA fluoresces under blue light appearing red in bulk tumour areas, pink around the margins, and disappears completely with diminishing tumour density, thus giving room for a high degree of ambiguity [16,34]. The use of 5-ALA allows for a more precise resection procedure and does not depend on damage to the BBB [16] in order to reach the tumour site [6]. Rather, it is dependent on upregulated cellular transport mechanisms leading to intracellular accumulation and is dependent on cellular metabolism and specific tumour microenvironment [35].

In GBM, use of 5-ALA is associated with an increase of 29% in CR and a significant reduction in residual contrast-enhancing tumour on postoperative MRI (a predictor for recurrence), when compared to white light only resections [6,19]. This translates into longer PFS, a reduced need for re-interventions, and a 3.05 month increase in OS [4,5,6,22,36]. Moreover, fluorescence can be detected beyond the margins of the contrast-enhancing tumour on MRI, therefore suggesting its use in visualising non-enhancing tumour [35]. Recently, several groups have begun to explore the use of 5-ALA with photodynamic therapy (PDT) in the management of GBM [37]. The PpIX (thusly 5-AlA) is not only fluorescent but has been shown to be phototoxic. Due to its high specificity of accumulation in the tumour, it has been suggested that PDT of the surgical resection cavity may further enhance survival in GBM patients [38]. Intra-operative PDT can target the tumour in infiltrating margins following 5-ALA FGS surgical resection to ensure CS [38]. A pilot clinical trial (NCT03048240), which has implemented this approach, has been initiated by University Hospital Lille, in collaboration with the Institut National de la Santé Et de la Recherche Médicale (INSERM, Paris, France). Briefly, 10 patients with GBM with complete surgical removal received 5-ALA FGS and intra-operative PDT in combination with current SOC postoperatively. After iMRI to assess the extent of surgical resection, the PDT was delivered at five fractions of 5 J/cm^2^. At an interim analysis of the patients, the 12-months progression-free survival (PFS) rate was 60% (median 17.1 months), and the actuarial 12-months OS rate was 80% (median 23.1 months), suggesting that 5-ALA PDT may help to decrease the recurrence risk by targeting residual tumour cells in the resection cavity [38].

Notwithstanding the ostensible successes of 5-ALA, there are drawbacks. Currently, the available microscopes to view fluorescence from 5-ALA operate under dark-field conditions [4] resulting in an inability to identify important neurological structures. Another shortcoming of 5-ALA is photobleaching, which is the reduced intensity of emission light with prolonged exposure to the activating light [4,22]. This is overcome under normal circumstances as new tissue is continually re-exposed throughout the procedure [22]. Furthermore, 5-ALA produces a 2D image meaning fluorescence can be missed by overhanging tissue, but this limitation, however, can be overcome by dissecting the tumour margin [22]. Skin sensitisation, moreover, requires the patient to avoid sunlight or direct artificial light for up to 24 h post-administration [19,22]. However, the use of 5-ALA in combination with other dyes or modalities has the potential to overcome its shortcomings. The 5-ALA may also be used in combination with FS to better illuminate tumour tissues. This combination appears orange-to-red in the parts where tumour is present, and green in normal tissue thus increasing sensitivity and specificity of tumour and yielding improved surgical resection margins [4,22]. Finally, iMRI, in addition to FGS with 5-ALA, can produce a GTR of up to 100% of contrast-enhancing tumour detailing the complimentary use of both modalities of imaging in GBM tumour resection [4]. In trials assessing the safety of 5-ALA, preoperative and postoperative KPS score, neurological status, hepatobiliary enzyme levels and blood count were generally unchanged [6,39]. This safety margin, combined with its efficacy in delineating tumour cells in high-grade gliomas has led to its approval for use in intra-operative imaging by the U.S. Food and Drug Administration (FDA) [35,40].

### 2.3. Indocyanine Green

Indocyanine Green (ICG) is a hydrophobic dye [4] which attaches to plasma proteins within blood vessels [19], and serves as a tool for both observing blood flow, and an aid for surgical guidance. It has an emission peak of 820 nm so is considered a near-infrared (NIR) agent [19,41]. The use of ICG results in lower tissue autofluorescence and deeper tissue penetration than 5-ALA. It is the only clinically approved NIR fluorophore [42] and is currently FDA-approved for use in ophthalmologic angiography and hepatic function assessment [43]. The ICG is excreted exclusively in bile and along with its non-specific nature and short in vivo half-life of 4 min [42], the current use in FGS of tumours is limited to hepatocellular carcinoma (HCC) [44].

Recent clinical trial data has shown its potential for intra-operative tumour/normal tissue classifications when used in conjunction with AI image analysis of tissue perfusion profiles [45]. For example, Cahill et al. (2021) demonstrated in 24 patients (11 with colorectal cancer CRC) that the wash-in kinetics of ICG (as analysed by AI) of normal and of cancerous tissue was significantly different and was able to determine the patients with CRC with a specificity in tumour detection of 95% and a sensitivity of 92%. A novel emerging technique for use in glioma surgeries is second-window ICG (SWIG) [46,47]. This approach involves a high-dose of ICG administered 24 h preoperatively and employs the enhanced permeability and retention (EPR) effect observed in tumours [46]. The EPR effect results from the abnormal vasculature and inadequate lymphatic drainage of cancers [48]. In a recent study [46], SWIG resulted in increased accumulation in HGGs (96% sensitivity) and rapid clearance from normal brain tissue. Nevertheless, the use of ICG in FGS is costly, and requires alternating use of white light and NIR, which requires separate display monitors to overlay tumour tissue fluorescence with conventional light [29]. Another limitation of ICG is the detection of false-positives by producing signals in areas of necrosis and inflammation [47,49] due to corresponding fluorescence patterns with Gadolinium-enhancing tissue [49]. Future studies should implement SWIG in the resection of GBM as this approach has the potential to overcome the shortcomings in current ICG use.

### 2.4. IRDye 800 CW

The NIR dye IRDye 800 CW emits at 805 nm. It is often conjugated with specific molecules aimed to specifically target cancer tissues. Cetuximab, an FDA approved monoclonal antibody that inhibits Epidermal Growth Factor Receptor (EGFR), is an example of a molecule that can be conjugated to IRDye 800 CW [4,18,30]. The EGFR is highly expressed in 50–70% [50] of GBMs and is, therefore, a promising target for diagnosis of GBM [22,29]. A first in-human trial of cetuximab conjugated IRDye 800 CW was conducted by Miller et al. (2018) [50]. Therein, two patients, with preoperative MRI scans showing contrast-enhanced tumours ranging from 1.5–8 cm in diameter, were enrolled in the trial. One patient received a low dose of the cetuximab-conjugate (50 mg), while the second patient received a high dose cetuximab-conjugate (100 mg). Viable tumour tissue was identifiable in the low dose patient with a sensitivity of 73.0% and a specificity of 66.3% (tumour vs. normal tissue). In the high dose patient, a sensitivity of 98.2% was achieved, with a specificity of 69.8% (CI 64.3–74.9). One of the major benefits of the NIR conjugate systems is the wide availability in many major surgery centres of the imaging infrastructure necessary to utilise this technology. This eliminates the need to train surgeons or purchase new imaging devices while rapidly implementing the technology.

The use of IRDye 800 CW conjugated to an affibody (ABY-029) in glioma surgery has also been investigated. Affibody molecules are synthetic peptides and non-immunoglobulin proteins, which can be modified for use in radioactive labelling. Therein, Samkoe et al. utilised the affibody ABY-029, which serves as an EGFR inhibitor [51] and a targeted fluorophore for intra-operative imaging. In a recent preclinical study [51], in the first hour post-administration in GBM tissue of mice, there was an 8- to 16-fold average increase in fluorescence visualised in the tumour relative to normal brain with fluorescence still present after 48 h [52]. The mean half-life for cetuximab is 4.7 days [53], and it was demonstrated that when in used conjugation with the NIR dye, this half-life was notably reduced to an average of 27 h across cohorts [54]. A long half-life translates to maximum uptake in tissue [54], however it also requires lengthened clearance from surrounding tissue [19,44], and an improved balance is therefore reached with the use of dyes in the NIR spectrum. Future studies should focus on reducing the prolonged accumulation in normal brain parenchyma while optimising imaging protocols.

## 3. Novel Dyes in Pre-Clinical Development

**Figure 2:**
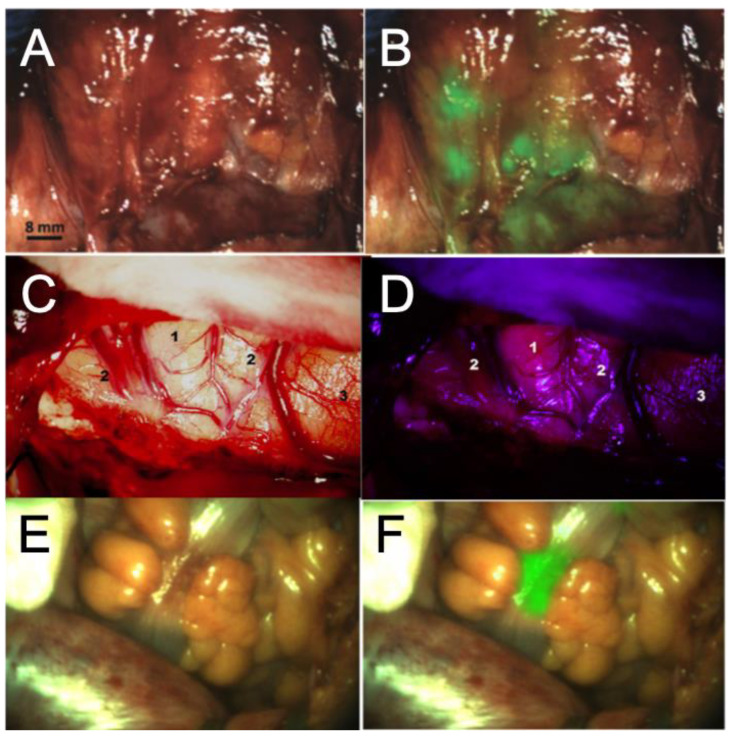
Pre-clinical assessment of novel dyes for Glioblastoma Fluorescence Guided Surgery (FGS). Representative images of novel dyes currently in pre-clinical assessment for use in Glioblastoma Fluorescence Guided Surgery (FGS). (**A**,**B**) Intra-operative detection of ovarian metastasis using a Folate-Targeted fluorescent probe. (**C**,**D**) Intra-operative detection of malignant glioma following IV injection of Hypericin. (**E**,**F**) Intra-operative fluorescence of a palpable colorectal tumour using an RGD conjugated agent. Images are shown under either white light (**A**,**C**,**E**), white light and fluorescence overlay (**B**,**F**), or under blue fluorescence. Images (**A)** + (**B)** reproduced with permission from Hoogstins et al. *Clin Cancer Res.* **2016**, *22*, 2929–2938. Images (**C**) + (**D**) reproduced with permission from Ritz et al. *Eur. J. Surg. Oncol.* (EJSO). **2012**, *38*, 352–360. Images (**E**) + (**F**) reproduced with permission from de Valk et al. *Ann. Surg. Oncology*. **2021**, *28*, 1832–1844.

Due to the limitations associated with currently available fluorophores, as outlined above, there is a continuing need to develop new strategies for fluorescence imaging as applied in neuro-oncology surgical applications. These strategies include the development of novel agents, as well as the adaption of new variants of existing probes, which may be implemented either alone or in combination with existing approaches (Figure 2). One approach to designing novel agents is to fluorescently tag known tumour targets, such as EGFR, HER2, and VEGF. These targeted fluorescence probes may then be employed for visualisation of tumours [55]. Table 2 shows novel fluorophores still undergoing preclinical trials. As discussed below, these probes aim to address the shortcomings of established fluorophores, such as non-specific fluorescence and unwanted adverse effects. 

### 3.1. Folate-Targeted FGS

The folate receptor (FR) is highly expressed in neoplastic cells, and can serve as a useful target for fluorescence probing [19]. Fluorescein iso-thiocyanate (Folate-FITC) has an emission wavelength of 520 nm, and is an example of one such FR-targeting fluorophore [19]. Once Folate-FITC binds to the folate receptor, endocytosis occurs slowly with the fluorescent conjugation persisting within the cell after 2 h [19]. Indeed, GBM tumours display a high expression of FR-α, rendering this receptor a potential target for brain tumour-specific fluorescence imaging [56,73]. Effective targeting of GBM via FR requires the consideration of other FR expressing components of the tumour microenvironment (TME), such as tumour-associated macrophages (TAMs) [56]. The TAMs constitute up to 50% of tumour bulk in GBM and have been shown to play an important role in tumour maintenance and progression [74]. However, FR is only moderately expressed within TAMs, presenting a challenge to target them specifically. It has been suggested to overcome this challenge through the use of recently developed Carbon nanosphere technology [56]. Carbon nanospheres (CSPs) are distinguished by their ability to cross the BBB and may improve the targeting of FRs specifically on GBM cells [56]. The CSPs along with an FR-targeting agent (F8) form CF8 [56], which has the combined ability to target FR-expressing cancer cells and TAMs across the BBB [56]. The CF8 may, therefore, serve as a selective target to FR-expressing glioma cells. Therefore, this BBB-crossing feature of CSPs overcomes a limitation of conventional FR-targeted delivery systems [56]. In this study investigating the use of CF8 as a dual drug delivery system to glioma cells and TAMs, the dye 1,1-dioctadecyl-3,3,3,3- tetramethylindotricarbocyanine iodide (DiR)-labelled CF8 was injected into glioma-bearing mice. There was increased accumulation of CF8-DiR in glioma tumours when compared to CSP-DiR or free DiR dye alone, and fluorescence was observed using an in vivo imaging system [56]. The findings from this preclinical study revealed clear benefits of using this dual targeting strategy for simultaneously targeting both FR-expressing tumour cells and TAMs in the tumour microenvironment. Indeed, this strategy may lead to improved tumour resection and increased patient survival.

### 3.2. Hypericin

Hypericin, a naphthodianthrone, is a strong lipophilic fluorophore [75] with an emission peak of 650 nm [18]. This agent has the potential to improve glioma diagnosis and treatment due to its higher photostability and penetration depth in comparison to 5-ALA [75]. In a pilot study to investigate its use in identifying High Grade Gliomas (HGG) [75], tumour tissue was clearly apparent from normal brain tissue. Hypericin appears red in areas where it is strongly fluorescent, as in the core of the tumour bulk, and appears pink in weakly fluorescent areas towards the margins where the tumour density is decreased, while normal tissue appears blue [75]. In a further study employing implanted C6 glioma cells in rats, hypericin selectively accumulated intracerebrally and maximum uptake was recorded 24 h following administration [57]. Sensitivity and specificity in differentiating tumour from healthy tissue ranged between 90–100% and 91–94%, respectively [75]. The performance of hypericin has the potential to be highly beneficial to patients with tumour recurrence. This is because clear tumour delineation is difficult, particularly at tumour margins, due to the infiltrative nature of GBM [75]. Nevertheless, the decision on what is considered strongly and weakly fluorescent is user-dependent [75], thus giving variable results, which can affect the EOR. 

### 3.3. RGD Conjugated Agents

The NIR fluorophores such as ICG are limited in their use due to their non-specific accumulation in tissue and their short in vivo half-life [44]. Nevertheless, more recent NIR bio-conjugated fluorophores have been developed to specifically target certain tumour types such as GBM [44]. Additionally, bio-conjugated fluorophores have a large molecular weight and, therefore, have a longer half-life. This greatly enhances their therapeutic uses in treating cancer, as this leads to prolonged accumulation in GBM tissue. However, to allow for clearance from normal tissue, there is an increased time delay experienced between administration of the fluorophore and the commencement of imaging [44]. To allow for more accurate intra-operative imaging at earlier timepoints, it will be necessary to find other approaches that enhance the target-to-background signal ratio. A plausible approach to achieve this would be using mechanisms of selective fluorescence quenching in background tissue. By first establishing the emitting potential of the fluorophore within the region of interest (ROI), and then quenching fluorescence in background areas, it will allow the issue of background clearance to be overcome [44].

Integrins are a potential target for bio-conjugated fluorophores with αvβ3 and αvβ5 highly expressed in GBM [21,60,76]. Integrins such as αvβ3, play a role in tumour angiogenesis and their upregulation is also associated with increased cancer growth and metastasis [44]. The tripeptide arginine-glycine aspartic acid (RGD) sequence can recognise and bind αvβ3 and αvβ5, and promotes cellular internalisation. Due to these characteristics, conjugates of the more stable cyclic variant c(RGDfk) are being investigated as selectivity enhancers for tumour therapies and diagnostics [44]. In a study investigating therapeutic targeting of integrins in cancer [76], cRGD has shown affinity towards integrins αvβ3 and αvβ5 in GBM [21,60,76]. Like cRGD, the peptide iRDG has affinity for high levels of αv integrins on the surface of tumour vessels. The iRDG peptide binds to αvβ3 and αvβ5 and is then proteolytically cleaved within the tumour to produce CRGDK/R [44,77]. The iRGD peptide also has an affinity for neuropilin-1 (NRP-1). Binding of the iRGD peptide to NRP-1 results in tumour tissue penetration and uptake, which is useful in drug delivery [77]. This tumour-specific targeted approach of RGD conjugates and the success in targeting cells for drug delivery can be translated for use in FGS of GBM as is done with RGD-conjugated fluorophores like IRDye 800 CW-RGD and cRGD-ZW800-1. In a transgenic GBM mouse model, the IRDye 800 CW-RGD dye specifically bound to the integrin receptors harboured on GBM cells and gave a detectable fluorescence [60]. In this study, conjugation of this NIR fluorophore (IRDye 800 CW) to the RGD motif did not impede the fluorescence activity of IRDye 800 CW, nor the integrin affinity of RGD [60]. Furthermore, the study was performed with the IRDye 800 CW dye, which is of 800 nm emission wavelength simultaneously with the 700 nm of the dye and reports showed no interference on the images [60]. This demonstrates the potential for combining dyes with varying emission wavelengths in future research to improve EOR in GBM surgery [60]. There were also no reports of effects from photobleaching.

cRGD-ZW800-1 is a cyclic pentapeptide RGD conjugated to the 800 nm NIR fluorophore, ZW800-1 [21]. Delineation of tumour tissue from normal brain was observed in a GBM-mouse model investigating with cRGD-ZW800-1 and when compared to IRDye 800 CW [21], the conjugate had a lower non-specific uptake in normal tissues. An increased dose of 30 nmol of cRGD-ZW800-1 resulted in no non-specific uptake in surrounding tissue [21]. This, therefore, has the potential to be the optimal dose of cRGD-ZW800-1 to achieve maximal safe resection in GBM surgery and future research needs to investigate the benefits of resection at this dose. Additionally, a TBR of 17.2 was achieved within 4 h in comparison to 5.1 with IRDye 800 CW [60]. Optimal fluorescence is achieved within hours of administration as opposed to days when compared to antibody labelled fluorophores, and overall targeting RGD-binding integrins has shown to be well-tolerated in preclinical studies [21]. Ultimately, these RDG conjugated compounds have promising potential to vastly improve FGS in GBM as they overcome tumour specificity issues, toxicity, and photobleaching. These compounds represent a new chapter for intra-operative agents in GBM, which will ultimately lead to improved surgical outcomes for patients.

### 3.4. Alkylphosphocholine Analogues (APCs)

The APCs are synthetic phospholipid ether molecules that selectively accumulate [62] in overexpressed lipid grafts of various tumours such as GBM [29]. The APCs are resistant to catabolic breakdown, which prolongs time in the tumour microenvironment, and translates to prolonged fluorescence in targeted tissue during surgical resection [29]. In a preclinical study comparing the selective accumulation of 5-ALA with fluorophore labelled APCs (CLR1501 and CLR1502), in five preclinical models of GBM (U251, two patient derived xenografts (PDXs) and three GBM stem cell PDX lines) [62], CLR1501 showed an accumulation and fluorescence profile similar to 5-ALA, while CLR1502 showed a clearer and more defined tumour to brain distinction using IVIS spectrum imaging, Fluobeam detection, and commercially available operative microscopes with appropriate fluorescence detection attachments. These data agree with previous studies, as CLR1502 contains a NIR cyanine fluorophore and light in this spectrum can effectively decrease light absorption in targeted tissue and the false positives of tissue autofluorescence [62]. The tumour-specific uptake of APC-conjugated fluorophores enables greater precision during GBM resection and a heightened potential for preservation of normal brain tissue. Nevertheless, it is noteworthy that practical limitations for these NIR compounds remain. Specifically, the use of NIR agents in surgery requires the tumour to be visualised on a separate monitor in a darkened operating theatre. As a result, surgeons cannot simultaneously visualise the resection field and instruments directly as they would in 5-ALA-guided surgeries.

### 3.5. NIR-AZA Compounds

**Figure 3:**
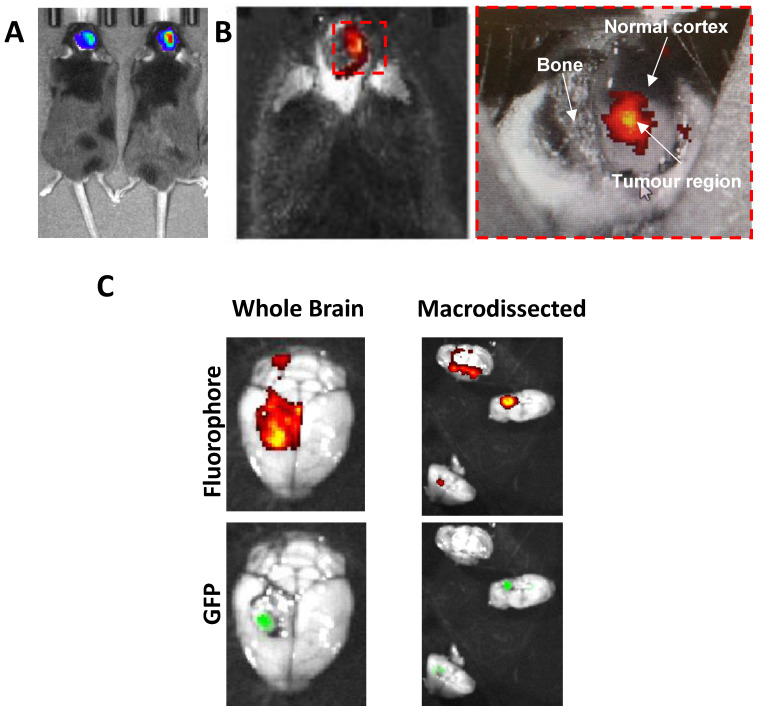
Preclinical assessment of a novel NIR-AZA Fluorophore. Mice were implanted with 2 × 10^5^ NFPp10a-GFP cells, and tumours allowed to develop for 14 days. Tumour growth was monitored by bioluminescence imaging. Subsequently mice then underwent a partial craniotomy to expose tumour and normal tissue. Fluorophore was then injected IV and mice fluorescently imaged for 90 min on IVIS Spectrum. (**A**) Representative image of mice showing location of implanted GBM tumour by bioluminescence. (**B**) Representative images of mouse post partial craniotomy illustrating exposure of normal and tumour tissue after fluorophore injection. (**C**) Ex-vivo imaging of whole and macro-dissected brain. As the tumour was also tagged with Green Fluorescent Protein (GFP), fluorescence imaging was also performed to confirm tumour location [Connor, Shiels et al., Unpublished data].

BF2-azadipyrromethene (NIR-AZA) is an NIR fluorophore class, developed by the authors, which has shown promise in FGS. Our research efforts have focused on the BF2-azadipyrromethene (NIR-AZA) fluorophore class due to excellent photophysical characteristics including substituent determined emission maxima between 675 and 800 nm, high quantum yields and exceptional photostability [20,78]. Furthermore, linking poly(ethylene-glycol) (PEG) units to NIR-AZA enhanced fluorescence emission. Photostability was also improved, with no light activation of O_2_ and no in vivo toxicity associated with the doses recommended for imaging [79]. The PEG plays a vital role in drug delivery by solubilizing and protecting the paired agent, which in this case is the fluorophore. It protects the fluorophore from the aqueous environment while extending the half-life [32]. Additionally, the bio-responsive NIR-AZA has off/on fluorescence switching controlled by the in vivo interconversion of phenol to phenolate [34,56]. Bio-responsive NIR-AZA can be used for real-time continuous imaging of cellular processes such as endocytosis, lysosomal trafficking, and cellular efflux [56]. With the photostability and long half-life, and the specific nature of bio-responsive NIR-AZA compounds, NIR-AZA compounds have the potential to be applied for use in targeted GBM imaging as they address the necessary shortcomings in established fluorophores currently in use for surgical resection of GBM. In recent unpublished work investigating the efficacy of the NIR-AZA agents in a mouse model of GBM by the authors (Figure 3), we demonstrate that following IV injection of the novel fluorophore, activity is only observed in the tumour bearing region of the brain. The implanted tumour (Nfpp10a mouse GBM cell line [80]) is additionally fluorescently labelled with green fluorescent protein (GFP) which can be detected via optical imaging. Figure 3B highlights the activity of the novel fluorophore which overlaps with the GFP fluorescence of the tumour, detailing the specificity of this novel compound. Further work is now required to investigate this agent as a robust intra-operative imaging agent.

## 4. Conclusions

Glioblastoma (GBM) remains one of the most aggressive tumours and a significant source of mortality and morbidity in adults. Current SOC aims to achieve maximum safe resection of neoplastic tissue while preserving healthy brain tissue. This is especially important in the eloquent regions where supramarginal resection is not possible without risk of neurological deficit [81]. However, delineation of normal from tumour tissue continues to be an ongoing challenge. Indeed, despite radiological tools like MRI and ultrasonography, which have proven capable of identifying tumour bulk and in some cases residual tumour pre- and post-operatively, use intra-operatively is met with several limitations, which has given rise to the use of fluorescence-guided surgery (FGS). Nonetheless, there is opportunity for improvement in surgical resection protocols with the use of FGS, specifically as many challenges persist in the development of targeted, effective, and safe fluorophores for use in this disease context.

Challenges in the development of fluorophores include a requirement for well-designed clinical trials to assess safety and efficacy. Within these trials, it is important that appropriate outcome measures are selected to assess performance. The GTR and PFS are often used as indicators of clinical outcome. However, overall survival or development of neurological symptoms may be more relevant clinical markers [18]. Further research is also required to investigate fluorophores, both alone and in combination, to determine the optimal dose and pre-imaging administration schedule to optimise their fluorescence profile and achieve maximal resection. Indeed, a combination of PET guided-surgical planning with intra-operative fluorescent agents, may provide improved resection margins compared to intra-operative agents alone. This combination has the potential to greatly improve the impact of both individual modalities on safe maximal surgical resection and, therefore, enhance the welfare and outcome of GBM patients [82]. The half-life of the fluorophore within the body must also be considered, as this will affect the clearance from the tissue surrounding the tumour. Finally, the technological advancements in neurosurgery do not obviate the need for a detailed consideration of neuroanatomy and rigorous pre-operative planning and monitoring during the procedure [83]. Critically, in the recent review by Mieog et al. [84], it was suggested that for FGS to succeed, target selection, imaging agents and their related detection machinery and their implementation in the clinic have to operate in synergy with each other. Only then will FGS truly improve patient outcomes. Furthermore it has been suggested that the favoured approach of FGS is specifically targeted fluorophores which allow specific accumulation in the tumour and its microenvironment, with biological conjugates leading the way [84]. These conjugates are molecules that target specific tumour epitopes (e.g., integrins or surface receptors). Therefore, the design of targeting tumours (tumour tuning) with a conjugated fluorophore has the potential to produce favourable results in GBM via reduction in residual tumour, which will translate to decreased tumour recurrence, GBM patient morbidity and mortality. Indeed as discussed in this review, one future direction for the development of novel fluorophores is the use of “switching fluorophores” i.e., probes that become fluorescentally active under particular tumour associated conditions, such as acidic pH of a tumour [85]. Switching fluorophores greatly enhances tumour selectivity and may increase the extent of tumour resection. Furthermore, these agents could be conjugated to tumour specific ligands like RDG peptides or other tumour specific markers (EGFRVIII for example). Overall, the field of FGS as applied in the neuro-oncology setting is rapidly evolving and expanding. Nonetheless, this FGS in neuro-oncology still needs to overcome some major hurdles before widespread implementation as a critical tool for improving surgical resection of GBM.

## Figures and Tables

**Table 1:** Status of Fluorophores in clinical trials.

Fluorophore	Chemical Family	Excitation Wavelength (nm)	Emission Peak (nm)	Mode of Action	Trial Number	Tumour	Aim/Result	Reference
**Fluorescein**	Fluorescein	460–500	510–525	Passive	NCT03752203	Paediatric Neurosurgical Tumours	Determine EOR of Intracranial and spinal lesions using Fluorescein Sodium	https://clinicaltrials.gov/ct2/show/NCT03752203 (accessed on 16 February 2022)
					NCT02691923 Phase 2	High grade glioma	Determine the diagnostic potential of Fluorescein through an operating microscope relate to (1) contrast enhancement on co-registered preoperative MR scans, (2) intra-operative ALA-induced PpIX fluorescence and (3) gold-standard histology obtained from biopsy sampling during the procedure.	https://clinicaltrials.gov/ct2/show/NCT02691923 (accessed on 16 February 2022)
**5-ALA**	Endogenous non-proteinogenic amino acid	400–410	635–710	Metabolic	NCT00241670 Phase 3	Malignant Glioma	29% more Complete Resections 6-month higher PFS	[6]
					NCT02755142 Phase 1/2	Malignant Glioma	100% Positive Predictive Value 10-fold increase in dose led to 4-fold increase in contrast between tumour and brain 20 mg/kg gave the strongest fluorescence	[23]
					NCT00752323 Phase 2	Malignant Astrocytoma	Determine the optimum dose and administration time of 5-ALA	https://clinicaltrials.gov/ct2/show/NCT00752323 (accessed on 16 February 2022)
					NCT02379572	Glioblastoma	Comparison of iMRI and 5-ALA on number of complete resections	
					NCT01128218 Phase 1,2	Malignant Glioma	Determine specificity and sensitivity of 5-ALA fluorescence	https://clinicaltrials.gov/ct2/show/NCT01128218 (accessed on 16 February 2022)
					NCT02191488 Phase 1	Low, and high grade gliomas, Menihgiomas, or metastases	Red-light excitation of PpIX revealed tumour up to 5mm below resection bed in 22 of 24 tumours already visualised with blue-light.	[24]
					NCT00870779 Phase 1	Low, and high grade gliomas, Menihgiomas, or metastases pituitary adenoma or metastasis	Determine degree of spatial correlation between local fluorescence recorded intra-operatively and co-registered conventional imaging obtained preoperatively via MRI and intra-operatively via ultrasound and operating microscope stereovision	https://clinicaltrials.gov/ct2/show/NCT00870779 (accessed on 16 February 2022)
					NCT01502280 Phase 3	Low-grade Gliomas	Intra-operative confocal microscopy identified 5-ALA tumour fluorescence at a cellular level in 10 consecutive patients.	[25]
					NCT01116661 Phase 2	Glioma	Mean CPpIX was higher in fluorescing samples than nonfluorescing samples. Visible fluorescence can be used in line with Quantitative PpIX analysis	[26]
					NCT02155452	Malignant Glioma	Study the heterogeneity of fluorescence within malignant gliomas by sampling tissues from variable areas within the same tumour	https://clinicaltrials.gov/ct2/show/NCT02155452 (accessed on 16 February 2022)
					NCT02119338	Recurrent glioma	Correlation of 5-ALA fluorescence in tumour tissue with pathological findings	https://clinicaltrials.gov/ct2/show/NCT02119338 (accessed on 16 February 2022)
					NCT02050243 Phase 1/2	CNS Tumour, Paediatric	Determine sensitivity of CNS in identifying paediatric CNS tumours and number of patients with associated side effects	https://clinicaltrials.gov/ct2/show/NCT02050243 (accessed on 16 February 2022)
**ICG**	Cyanine	780	800–830	Passive	NCT03262636 Phase 1	Primary and Recurrent Brain Tumour	Determine the sensitivity of ICG uptake and expression in identifying autonomic nervous system tumours	[27]
**BLZ-100**	Chlorotoxin peptide + ICG	730–785	760–841	Targeted	NCT02234297 Phase 1	Glioma	Determine safety of BLZ-100 in adult patients with glioma undergoing surgery.	[28]
					NCT02462629 Phase 1	Central Nervous System (CNS) Tumours	Determine safety of BLZ-100 in paediatric patients with CNS Tumours	https://clinicaltrials.gov/ct2/show/NCT02462629 (accessed on 16 February 2022)
**Panitumumab-IRDye 800 CW**	IRDye 800 CW	775	789–795	Targeted	NCT04085887 Phase 1/2	Paediatric brain neoplasms	Determine the safety and efficacy of Panitumumab-IRDye 800 CW in removing suspected tumours in paediatric patients	https://clinicaltrials.gov/ct2/show/NCT04085887 (accessed on 16 February 2022)
**ABY-029**	IRDye 800 CW	775	789–795	Targeted	NCT02901925 Phase 1	Recurrent Glioma	Determine if microdoses of ABY-029 lead to detectable signals in sampled tissues with an EGFR pathology score ≥ 1 based on histological staining.	https://clinicaltrials.gov/ct2/show/NCT02901925 (accessed on 16 February 2022)
**LUM015**	Cy5	633–647	675	Metabolic	NCT03717142	Low grade glioma, Glioblastoma	Determine the safety and efficacy of LUM015 for imaging low grade gliomas, GBM and tumour metastasis to the brain	https://clinicaltrials.gov/ct2/show/NCT03717142 (accessed on 16 February 2022)
**Demeclocycline**	Demeclocycline	402	535	passive	NCT02740933	Brain Tumour	Determine if fluorescence is observable via confocal microscopy.	https://clinicaltrials.gov/ct2/show/NCT02740933 (accessed on 16 February 2022)
**BBN-IRDye 800 CW**	**IRDye 800 CW**	775	789	Targeted	NCT02910804	Glioblastoma	Determine the efficacy of BBN-IRDye800 CW in GBM patients	https://clinicaltrials.gov/ct2/show/NCT02910804 (accessed on 16 February 2022)
					NCT03407781	Lower grade Glioma	Determine the efficacy of BBN-IRDye800 CW in lower grade glioma patients	https://clinicaltrials.gov/ct2/show/NCT03407781 (accessed on 16 February 2022)

Each fluorophore was categorised into passive (non-selective accumulation in tissue), targeted (selective binding to a specific molecule in the tissue) and metabolic (requires metabolic process for activation) n/a: not applicable, results not published.

**Table 2:** Fluorophores undergoing preclinical evaluation.

Fluorophore	Chemical Family	Excitation Wavelength (nm)	Emission Peak (nm)	Mode of Action	Tissue Type	Result	Reference
**CF8 − DiR**	CSP + F8 + DiR	750	782	Targeted	Glioma in mice	Folate-targeted CF8-DiR showed a significantly higher accumulation than CSP-DiR. Free DiR dye remained localised in injection point showing accumulation was due to conjugation with CF8.	[56]
**Hypericin**		510–550	590–650	Passive	Glioma in rats	Tumour Background Ratio (TBR)s of 6 and 1.4	[18,57]
**Cetuximab-IRDye 800 CW**	IRDye 800 CW	775	789–795	Targeted	Orthotopic mice GBM	87% luciferase signal reduction compared to 41% with white light.	[58]
**Panitumumab-IRDye 800 CW**	IRDye 800 CW	775	789–795	Targeted	GBM in mice	30% higher TBR when using Panitumumab-IRDye 800 CW than 5-ALA	[59]
**IRDye 800 CW-RGD**	RGD Conjugate + IRDye 800 CW	775	789–795	Targeted	Mice Glioblastoma (GBM)	Renal clearance of IRDye 800 CW-RGD. The dye selectively binds to Integrin receptors on GBM tissue. TBR of 79.7 ± 6.9 in GBM	[60]
**Cyclic-RGD-PLGC (Me)AG-ACPP**	RGD Conjugate + Matrix Metalloproteinase (MMP-2)	620	670	Targeted	GBM Cells	Dual targeting improved uptake compared to either cRGD or MMP-2 alone. TBR of 7.8 ± 1.6 in GBM	[61]
**cRGD-ZW800-1**	RGD Conjugate	750–785	800	Targeted	GBM cell lines	36% more fluorescence signal recorded in comparison to unlabelled cRGD	[21]
**CLR1502**	Alkylphosphocholine (APCs) Analogues	760	778	Metabolic	Glioma in mice	TBR of 9.28 ± 1.08)	[62]
**CLR1501**	Alkylphosphocholine (APCs) Analogues	500	517	Metabolic	Glioma in mice	TBR of 3.51 ± 0.44 on confocal imaging; 7.23 ± 1.63 on IVIS imaging	[62]
**Chlorotoxin:Cy5.5**	Cyanine5.5	633	694	Targeted	Glioma-bearing mice	Mice injected with Chlorotoxin: Cy5.5 a 15-fold higher TBR at day 1 in comparison to mice with Cy5.5 alone	[63]
**Angiopep-2-Cy5.5**	Cyanine5.5	660–680	694	Targeted	GBM in mice	Tumour to normal fluorescence ratio (TNR) of 1.6 and 63% higher intracerebral uptake than PEG-Cy5.5, tumour margin was delineated non-invasively in vivo	[64]
**PEG-Cy5.5**	Cyanine5.5	650	665	Passive	GBM in mice	TNR of 1.1	[64]
**DA364**	RGD Conjugate	675	694–720	Targeted	GBM in mice	TBR of 5.14	[65]
**Methylene Blue**		642	688–700	Passive/Metabolic	Patient samples of Gliomas	Sensitivity and specificity of 95% and 100% respectively. Dye-enhanced multimodal confocal microscopy shows architectural and morphological features with similar quality to haematoxylin and eosin (H & E)	[66]
**PARPi-FL**	Inhibitor of the DNA repair enzyme PARP1	503	525	Targeted	GBM in mice	PARPi-FL showed low toxicity, high stability in vivo, and accumulates selectively in glioblastomas due to high PARP1 expression	[67]
**CH1055**	NIR-II	750	1055	Passive	Brain tumours in mice	Tumour was detected at depths of 4 mm.	[68]
**Anti-EGFR Affibody-IRDye-800 CW**	IRDye 800 CW	720	730–900	Targeted	GBM cell in mice	The small (6.7 kDa) protein Anti-EGFR Affibody was observed at high levels in outer edges of the tumour	[69]
**SDF-1-IRDye-800 CW**	IRDye 800 CW	685 and 785	702 or 789	Targeted	GBM cells	Fluorescence persisted for up to 4 days in-vivo	[70]
**IRDye800 CW-AE344 (uPAR)**	IRDye 800 CW	740 nm	850 nm	Targeted	Orthotopic GBM in mice	TBR above 4.5 between 1 to 12 h post injection	[71]
**VEGF labelled IRDye-800 CW**	IRDye 800 CW	675 and 745 nm	800 nm	Targeted	Mouse models of ovarian, breast and gastric cancers	TBR of 1.93 ± 0.40 on day 6 post administration	[72]
**EGFR2 labelled IRDye-800 CW**	IRDye 800 CW	675 and 745 nm	800 nm	Targeted	Mouse models of ovarian, breast and gastric cancers	TBR of 2.92 ± 0.29 on day 6 post administration	[72]

Each fluorophore was categorised into passive (non-selective accumulation in tissue), targeted (selective binding to a specific molecule in the tissue) and metabolic (requires metabolic process for activation).

## Data Availability

No new data were created or analyzed in this study. Data sharing is not applicable to this article.
